# Exclusive Breastfeeding and Its Determinants in Yaoundé, Cameroon: A Retrospective Survival Analysis

**DOI:** 10.1155/2022/8396586

**Published:** 2022-08-31

**Authors:** Gloria Akah Ndum Okwen, Esron Daniel Karimuribo, Helena Aminiel Ngowi, Edith Nig Fombang

**Affiliations:** ^1^College of Veterinary Medicine and Biomedical Sciences, P.O. Box 3015, Morogoro, Tanzania; ^2^Department of Veterinary Medicine and Public Health, Sokoine University of Agriculture, Tanzania; ^3^SACIDS Foundation for One Health, Sokoine University of Agriculture, P.O. Box 3297, Morogoro, Tanzania; ^4^National School of Agro-Industrial Science, P.O. Box 455, Cameroon; ^5^Department of Food Science and Nutrition, University of Ngaoundere, Cameroon

## Abstract

Exclusive breastfeeding (EBF) of infants for the first six months of life is a global public health goal that is linked to the reduction of morbidity and mortality in infants, especially in low middle-income countries. In low middle-income countries like Cameroon, it is realistic that compliance with EBF can significantly reduce the burden of under five mortality rate. The purpose of this study was to assess adherence and determinants influencing the duration of exclusive breastfeeding in Yaoundé, Cameroon. Data was collected through a mixed method and systematically through a retrospective survival analysis approach where a total number of 503 randomly selected individuals in Yaoundé, Cameroon, participated in the study. Data was collected between November 2019 and May 2020. A Cox proportional hazard modelling and Kaplan-Meier analysis were employed to identify prognostic factors affecting survival time defined as the duration, in months, from birth until the time of stopping EBF. The average time for nursing mothers to practice EBF was 3.61 ± 0.010 months. This study found that more than 90% of mothers were aware of the importance of compliance with EBF but only 38% practiced EBF and 62% of mothers could not adhere to EBF recommendations. Factors that influence compliance with EBF included a mother being married (HR: 0.70; 95% CI = [0.55-0.89], *P* =0.003) which was a protective factor while mother's tertiary education (HR: 1.43; 95% CI = [1.11-1.84], *P* =0.005) was a risk factor with non-compliance with EBF when compared to those with basic or no formal education. The Kaplan-Meier curve indicated that as time goes on, babies are less likely to be exclusively breastfed after a specific time period within two and three months. This implies that the chance for a baby to remain exclusively breastfed after five months is 74.3% (0.74). Continuous sensitization and enforcement measures are recommended to promote EBF.

## 1. Introduction

The World Health Organization [1] defines exclusive breastfeeding (EBF) as the procedure whereby infants receive only breast milk from a mother or wet nurse expressed to a child, with no other liquids or solids inclusive not even water for the first six months with the exception of oral rehydration solutions, syrups of vitamins and minerals, or medication. Exclusive breastfeeding of infants for the first six months of life is considered a global public health goal that is linked to the reduction of morbidity and mortality in infants especially in low middle-income countries [2]. International agencies and policy makers adopted a declaration in August, 1990, which affirmed that all infants should be exclusively breastfed from birth till four months of age. Subsequently, the WHO recommendations amended the breastfeeding declaration to six months in 2001 [3, 4]. Global public health recommendation by the WHO and the United Nations Children's Fund (UNICEF) requires infants to receive only breast milk for the first six months of life coupled with lactation and other complementary food for up to two years or beyond [5, 6]. Knowledge about breastfeeding recommendations is important as it may influence breastfeeding practices especially for first-time mothers [6]. Globally, approximately 34.8% of infants are exclusively breastfed for the first six months of life [7]. Exclusive breastfeeding is the first prized gift from mother to the baby that exists to benefit the baby, mother, and community [8, 9]. Exclusive breastfeeding has numerous benefits in terms of providing energy, protein, water, and other nutrients required for the development of an infant [10]. Other benefits of EBF include growth improvement as well as enhanced development of the immune system and cognitive ability of children [11]. Breast milk carries antibodies from the mother that helps in combating diseases and in addition helps prevent future harm and reduces spending on future health care [12, 13]. More so, breast milk also provides a healthy weight balance thereby reducing children's risk of becoming overweight [5, 14]. Evidence-based research has proven that some deaths can be prevented through proper breastfeeding methods where improved lactation rates can save globally over 800,000 children under five years annually [9, 15, 16]. Due to these benefits, the WHO and UNICEF recommend that all mothers should breastfeed their children exclusively for the first six months and thereafter continue to breastfeed for as long as the mother and child desires, with appropriate and sufficient weaning food included after six months of life [4, 11]. Non-compliance to adequate lactation practices is a public health threat and concern with major causes of deaths and morbidity in infants and young children [17]. Furthermore, failure to exclusively breastfeed for the first six months of life can result in 1.4 million deaths and 10% of disease burden in children younger than five years according to Black et al. and Gejo et al. [7, 18]. When an infant is inadequately breastfed, the child becomes more exposed to risk factors and health complications such as frequent gastrointestinal infections and poor brain development coupled with lower and upper respiratory tract infections [17, 19]. Approximately 41% of under 05 death's occurrence in Sub-Saharan Africa (SSA) is linked to inadequate breastfeeding in combinations with other diseases which causes about 30% of diarrhea deaths and 18% of acute respiratory deaths [1, 20]. Promoting proper feeding practices is one of the main programmed areas that the Department of Nutrition for Health and Development in Cameroon focuses on by providing counselling courses and guidance for the protection, promotion, and support of infant and young child feeding [21]. Promoting and striving to increase the trends of EBF in Cameroon are also done by implementing the National Breastfeeding Sensitization Plan by the Ministry of Public Health Cameroon (2016) in partnership with WHO in Cameroon. Exclusive breastfeeding is a well-acknowledged and cause-effective intervention against malnutrition-related illnesses in children [17]. Despite the documented importance and advantages of practicing EBF, the practice is still not widespread with an unsatisfactory level recorded across the globe and most especially in developing countries [18, 22]. More is yet to be done to narrow this gap and sustain EBF practices. Still, the associated factors vary within different countries and even within the same country where a gap still exists [22]. In Cameroon, about 92% of children less than six months old are exposed to breastfeeding, but of these, less than 50% are exclusively breastfed for six months and about 88.8% receive colostrum [7, 21]. This study was designed to assess adherence and to identify determinants influencing the shortened time to exclusively breastfed in Yaoundé, Cameroon, using a survival analysis approach. Interventions such as maternity leave and nursing breaks for working mothers were introduced although they are not fully implemented where only 42 countries (23%) meet or exceed the recommendation of 18 weeks' leave for breastfeeding mother [23]. Findings from this study are needed to provide and update information on the current situation and also make recommendations on what can be done to comply with the six months exclusive breastfeeding plan as recommended by the WHO [24].

## 2. Methodology

### 2.1. Study Area

This study was conducted in the urban city of Yaoundé in Cameroon. Yaoundé is located in the southern part of the country which has an elevation of about 726 meters above sea level and a total surface area of 180km^2^. The city is situated at 3°52′N and 11°31′E. Yaoundé is the second largest city in the country, after Douala, with an estimated population of 2.8 million inhabitants [25]. The average temperature in Yaoundé varies from 18.9°C to 30.6°C and rarely below 16.7°C or above 32.8°C.

### 2.2. Study Design

This was a hospital-based cross-sectional study conducted among 503 mothers with babies aged between six and ten months. Data was collected through a mixed method and systematically through a retrospective survival analysis approach where a total number of 503 randomly selected individuals in Yaoundé, Cameroon, participated in the study. A retrospective survival analysis approach was adopted in order to determine time to the occurrence of an unwanted event of interest (stopping of EBF). The prognostic factors, defined as factors which contributed to an abrupt cessation of EBF, were identified. During the study, the outcome variable was defined as the duration of months, from birth to the occurrence of cessation of EBF before the recommended six months. Censoring was considered when the child under consideration reached six months without experiencing the event of interest.

### 2.3. Sample Size Estimation

The sample size was determined using the Fisher formula: *S* = *Z*^2^∗*P*∗(1 − *P*)/*M*^2^ at a given prevalence rate of 0.452 with a confidence level of 95% and marginal error of 0.05. The estimated sample size was 381 participants. An additional 32% was added to the sample size giving a total of 503 participants from whom data was collected to have a greater statistical power to detect significant difference.

### 2.4. Survival Analysis Approach

It is a set of statistical methods used to analyze data in which the time until the event of interest occurs. The response is often referred to as a failure time, survival time, event time, and censoring [26, 27]. Censoring occurs when some information about a subject's event time is available but the exact event time is unknown. Generally, three reasons might account for censoring to occur: a subject does not experience the event before the study ends, a person is lost to follow-up during the study, and a person withdraws from the study [27]. Failure occurs when the event of interest occurs. For the purpose of our study, failure occurs when mothers stop exclusive breastfeeding of their babies before six months while censoring occurs when mothers do not experience the event of interest which is stopping EBF before the first six months of an infant's life.

### 2.5. Sampling Procedure

A simple random sampling technique was carried out. The first participant in the study was chosen at random and interviewed for about 10 to 15 minutes. Structured questionnaires were used to interview participating mothers by adopting a retrospective memory based on the past event of interest. Babies ranging from six to ten months were the ideal age group for recruitment into the study in order to minimize recall bias. The vaccination unit was the ideal location since babies had a routine vaccination dose to be administered at various age groups where data collection was of interest. Babies who recently turned six months were the main target in the study as they had to attend clinic at the vaccination unit for vitamin A and the first dose of Influenza immunization. The next target was babies who recently turned nine months old since they had a routine free immunization vaccine of MMR (Measles, Mumps, and Rubella) and a yellow fever vaccine to be received at the vaccination unit.

### 2.6. Inclusion Criterion

All nursing mothers attending antenatal clinic and vaccination unit in Yaoundé were included after their consent and approval of participating in the study. Breastfeeding mothers were between the age group of 18 and 45 years with babies less than 11 months who attended consultation and vaccination units. Two sets of twin babies were all handled separately and independently as they could record different caseation time to EBF.

### 2.7. Exclusion Criterion

Babies below six months old and babies from 11 months old and above were excluded from the study. Nursing mothers who willingly objected to be part of the study as well as babies whose biological mothers were not present during vaccination were excluded from the study. Confounders included babies with deceased mothers at birth, but their respective foster parents were interviewed on the baby's breastfeeding status. No disability case of a baby was encountered or visibly detected during the study.

### 2.8. Data Collection

Data was collected between November 2019 and May 2020 through formal interviews guided by structured questionnaire that was pre-tested before use. The questionnaire recorded key variables such as time to adherence to EBF as a dependent variable as well as other independent variables including maternal age, education level, employment status, marital status, antenatal care, immunization, colostrum intake, reasons for cessation of EBF, and baby developed diarrhea or any respiratory tract infection. Interviews were done individually and independently. The questionnaires comprised of both open and close-ended questions that were pretested after which probe questions were added to elicit clear responses. Responses from mothers were complemented by recording information available on the baby's vaccination card such as the baby's age, sex, and weight. Participant's consent was obtained and the interview session was recorded.

### 2.9. Data Analysis

A dataset was generated from the data collected using the questionnaire. Quantitative data was analyzed using SPSS version 21.0 (Statistical package for social science) and STATA Version 16 for statistical analysis. A Kaplan-Meier curve plotting was carried out for the survival probability. A Cox proportional hazard regression analysis was also used to investigate and identify different prognostic factors including assessment of their effects on the survival time. The relationship between the outcome variable and prognostic factors was analyzed (using univariable analysis) and corresponding *P*-values recorded. Those which qualified (*P* < 0.05) were considered being significant to the outcome variable for the next step of multivariable analysis and were fitted together using backward elimination approach. The final model included all prognostic factors which appeared significant at *P* < 0.05 or had significant biological significance for retention in the final model. For qualitative analysis, audios were verbatim transcribed and manually coded on Microsoft Word. Themes were developed and major exemplar quotations selected for each theme are presented in the “Results” section.

### 2.10. Ethical Consideration

Ethical clearance was obtained from the Regional Center Ethics Committee for Human Health Research at the Ministry of Public Health Yaoundé Cameroon in October 2019. All participants gave their consent to participate in the study and researcher signed and complied with confidentiality requirement throughout the data collection and processing period. Confidentiality was ensured at all levels since mothers were interviewed independently in a confined vaccination room with only staff members of the vaccination unit present.

### 2.11. Limitations

This study is not a representative of the whole Cameroon; hence, results of this work cannot be generalized to the entire country. However, the findings are still very informative about the current trend of EBF. Like any other study, due to limited resources, this study might have excluded other factors likely to influence EBF in Cameroon. In addition, long recall period for mothers could influence the validity of their responses.

## 3. Results

### 3.1. Demographic Distribution and Characteristics of the Mothers Who Participated in the Study

The age of mothers who participated in this study ranged from 18 to 44 years while; for babies, their age ranged from six to ten months. Approximately 70% of nursing mothers within the study were legally married or cohabiting. About 69% of respondents within the study reported to had attained a higher (tertiary) level of education as summarized in Table 1. Only 38% of nursing mothers practiced EBF and 62% of mothers could not adhere to EBF recommendations. Knowledge about breastfeeding recommendations was received by more than 90% of nursing mothers who attended ANC. It was observed that as time goes on, the probability to exclusively breastfeed decreased.

### 3.2. Demographic Distribution and Characteristics of Babies Who Participated in the Study

The average age group of participating babies within the study was six months old. During the study, approximately 26.4% of babies contracted diarrhea and 6.2% contracted respiratory problems as represented in Table 2.

### 3.3. Prognostic Factors Associated with Duration of Exclusive Breastfeeding

Prognostic factors that were significantly associated with the duration of EBF included mother's marital status and educational level. It was found that a mother who was married was more likely to comply with EBF (HR: 0.70; 95% CI = [0.55-0.89], *P* =0.003) when compared to a mother who was not married. With respect to the influence of level of education, a mother who had attained a tertiary education was more likely not to comply with EBF (HR: 1.43; 95% CI = [1.11-1.84], *P* =0.005) when compared with the mother with basic or no formal education as summarized in Tables 3 and 4. However, this study found that a nursing mother's employment status did not influence EBF duration (*P* value =0.425>5%) as shown in Table 4 but it was retained in the final model due to its biological importance and evidence from previous studies.

### 3.4. Kaplan-Meier Survival Analysis

**Figure 1:**
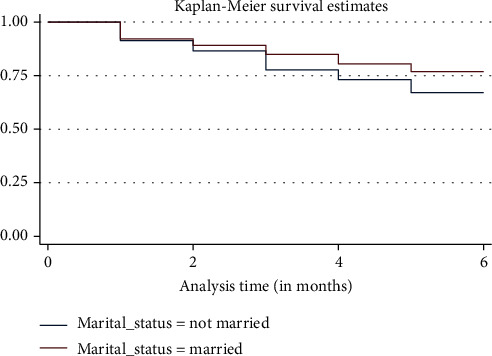
Survival function (Kaplan-Meier survival curve).

The Kaplan-Meier curve indicated that as time goes on, babies are less likely to be exclusively breastfed, more so after the second and third months. Thus, the chances for a baby to remain exclusively breastfed after five months is less than 75% for unmarried mothers as indicated in Figure 1.

### 3.5. Determinants to Nonexclusive Breastfeeding Based on Open-Ended Responses from Mothers

Our findings revealed that initiation and duration to EBF were influenced by some prognostic factors such as social and cultural attitude, availability of breast milk substitutes, mode of delivery, and unavailability of nursing mother. Exemplar quotes selected from themes of why some mothers did not practice EBF were:

“I didn't practice EBF after child birth because, I gave birth through caesarean section which inhibited my breast milk flow and for this reason I had to introduce my baby to artificial milk” Mother, Vaccination Unit Chantal Biya Foundation

“I stopped practicing exclusive breastfeeding because my baby used to scream a lot which was an indication to me that he needed more than just breastmilk and I had no other option than add up with artificial milk” Mother, Consultation Unit Chantal Biya Foundation.

“Some babies deliberately refuse breast milk no matter how you force them to eat for example my girl child is so selective to feeding” Mother, Vaccination Center Chantal Biya Foundation.

“Because I leave very early for work and come back late I can't consistently breastfeed my baby and I don't feel comfortable expressing breast milk for my baby because I believe that my baby' nanny will not hygienically handle it” Mother, Vaccination Unit FCB.

“My boy child cannot get satisfied on breast milk alone, he usually cries a lot when I give him only breast milk. So I decided to add up with cereals and fruit juice to make him healthy and satisfied” Mother, Consultation Unit Chantal Biya Foundation.

### 3.6. Misconception about Exclusive Breastfeeding

While some mothers reported factors that challenged them from practicing EBF, some mothers had some misconception about EBF practices. Misconception about EBF was discovered to be associated to some cultural practices and lack of proper knowledge about EBF. Exemplar quotes selected were:

“In my tradition a child cannot survive only on breastmilk alone for six months, you must mix it with something to make the bones of the baby very strong” Mother, Vaccination Center Chantal Biya Foundation.

“Breastmilk alone is very light weighted, the baby cannot be feeding on it alone, Yes, it is true that breast milk has good nutrients but it is not heavy enough to support the baby for long” Mother, Vaccination Chantal Biya Foundation.

## 4. Discussion

This study provided an overview on the determinants of EBF using the survival analysis approach in Yaoundé, Cameroon. It aimed at assessing the adherence and determinants influencing the shortened duration of exclusive breastfeeding among breastfeeding mothers in Yaoundé. Our findings highlight that the prevalence rate of EBF was at 38%. Previous studies conducted in Yaoundé, Cameroon [28] revealed an EBF rate of only 15% whereas other findings conducted in Yaoundé documented that about 42.5% of babies were exclusively breastfed for six months [12]. With reference to past studies conducted and the present study, much room for improvement is needed to reach the global breastfeeding target of 50% [29]. Despite the global and local efforts to promote exclusive breastfeeding practices, the rates are still below the recommended standards. Most first-time mothers fall victim to early cessation of EBF which is commonly substituted with artificial milk, water, juice, and other liquid food supplement [30]. According to UNICEF, if more efforts are provided, countries might meet up to the global target of at least 50% prevalence rate of EBF by 2025 and a target of 70% by 2030. A mother's decision to exclusively breastfeed her baby is influenced by a variety of factors. While most women are aware that breastfeeding is the best source of nutrition for infants, they often lack knowledge regarding the numerous health benefits or reduction in health risks that occur through breastfeeding. This lack of knowledge inhibits mothers from properly weighing the advantages and disadvantages of breastfeeding to make an informed decision [31]. In addition, findings from this study revealed that EBF was significantly common among married mothers than unmarried mothers. This pattern of results is also consistent with past evidence-based studies conducted in the United States of America with 25,197 telephone interviews of breastfeeding mothers; it was observed that children living with both parents were more likely to have been exclusively breastfed (80.4%) than children of other types of families [32]. Furthermore, exclusive breastfeeding was not common among higher educated women. Similar patterns were also seen in wealthier, better-educated, and urban women in other low middle-income countries [23, 33]. It is essential to note that this class of women perceived breast milk substitutes as modern and prestigious while associating EBF with being poor and unsophisticated [23, 33]. Our findings further revealed that the probability for a baby to be exclusively breastfed for six months is not significantly influence by the employment status of the mother. Notwithstanding, past studies had proven a significant difference between these two groups. Past studies conducted [34] revealed that the duration of EBF is higher among unemployed mothers where mothers who are unemployed also practice EBF as the best option of cost-effective practice [34]. Income earning also goes as a boosting factor for artificial milk purchase, thereby widening the gap of nonexclusive breastfeeding. It is also noted that the general decline in breastfeeding is also correlated to the production and availability of synthetic breast milk and its substitutes [16, 35]. This study also revealed that water was the main factor altering EBF for six months as most nursing mothers supplemented the baby's breastfeeding pattern with water to quench their thirst out of the grip of fear. Most breastfeeding mothers perceived that a baby needed to drink water from their tender age for healthy growth. Other supplements that interrupted EBF were cereals, corn porridge, fruit juice, and bananas. More so, some babies could not receive only breast milk from the first day of birth because some expecting mothers gave birth through the cesarean section and preterm delivery which did not stimulate enough milk production after child birth. This therefore prompted some mothers to involuntary practice mixed feeding. Improved counselling and sensitization plan on the benefits of EBF by peer breastfeeding counsellors or health care providers will go a long way to narrow the gap of nonexclusive breastfeeding. This strategy will bring about behavioral changes for some mothers not adhering to EBF practices. A study has proven that counselling and moral support offered by health care providers and peer counsellors can strengthen breastfeeding initiation, duration, and a positive outcome for EBF rates [36]. Based on evidence, the longer the breastfeeding duration, the lower the risk to disease exposure. Determinants to nonexclusive breastfeeding in this study were observed to be social and cultural attitude, availability of breast milk substitute, unavailability of nursing mother, assisted, and preterm birth. A recent study also indicated similar patterns where assisted delivery and long hospital stays, poor maternal health, and preterm delivery could result to inappropriate or late breastfeeding [23]. In addition, inadequate support, especially in the first weeks after birth, and anticipation of breastfeeding difficulties are some common reasons for breastfeeding cessation. More so, mothers who do not successfully breastfeed in their first pregnancies are less likely to attempt breastfeeding in subsequent pregnancies [23]. This study provides an overview on the current trends of EBF in Yaoundé, Cameroon.

## 5. Conclusion

Despite the implementation of the National Breastfeeding Sensitization Plan by the Ministry of Public Health Cameroon (2016) in partnership with WHO in Cameroon, EBF rates in Cameroon are still below satisfactory levels [21]. Findings from the study revealed that above 90% of mothers were knowledgeable about EBF benefits and equally attended an antenatal clinic. Notwithstanding, about 38% of mothers exclusively breastfed their babies for six months meanwhile 62% of mothers could not adhere to EBF practices. If much effort is vested upon nursing mothers through counselling and sensitization plans on the benefits of practicing EBF, the gap of EBF will be narrowed and the prevalence rate of EBF shall increase in subsequent years. Counselling and sensitization goals can be achieved through ANC visits. It was noted that prognostic factors which were significant to determinants of EBF durations were the mother's level of education and marital status. This study provides an overview on the current trend of EBF practices in Yaoundé. In addition, the findings present evidence that EBF rates in Cameroon are still below the satisfactory level and need to be improved. Exclusive breastfeeding remains the best approach for protection against numerous diseases. Therefore, we suggest that future studies should aim at rolling out interventions to narrow this gap.

## 6. Recommendations

Based on this finding conducted in Yaoundé, Cameroon, the study recommends the following: (i) Reformation of some national working policies for nursing mothers in some demanding sectors of employment, such as offshore mining and outstation diplomatic services. Revision of some policies will lead to flexibility and time to breastfed due to our qualitative analysis which indicated that the nature of their job hindered them from practicing EBF. (ii) Reinforce prenatal sensitization on the benefits of EBF for both mother and child. (iii) The state should train peer breastfeeding counsellors to carry out community-based counselling for knowledge brokering on EBF in their convenient language. (iv) Offer more counselling and training sessions on breast milk expression to support mothers establish sufficient milk supply. (v) Recommend mothers to increase frequency of breastfeeding to maintain milk supply.

## Figures and Tables

**Table 1:** Demographic distribution and characteristics of mothers (*N* =503).

Variable	Level	Number	Percentage
Employment	Employed	273	54.3
	Unemployed	230	45.7

Education level	Basic/no formal education	156	31.0
	Tertiary or higher	347	69.0

Marital status	Married/cohabitating	356	70.8
	Not married	147	29.2

Practiced EBF∗	Yes	191	38.0
	No	312	62.0

Practiced breast Milk expression	Yes	200	39.8
	No	303	60.2

Attended ANC ∗	Yes	501	99.6
	No	02	0.4

ANC: antenatal clinic; EBF: exclusive breastfeeding.

**Table 2:** Demographic distribution and characteristics of babies (*N* =503).

Variable	Level	Number	Percentage
Sex	Male	241	47.9
	Female	262	52.1

Colostrum intake	Yes	464	92.2
	No	39	7.8

Contracted diarrhea During the study	Yes	133	26.4
	No	370	73.6

Developed respiratory problems during the study	Yes	31	6.2
	No	472	93.8

Babies ages	6 months	224	44.5
	7 months	54	10.7
	8 months	16	3.2
	9 months	197	39.2
	10 months	12	2.4

Worthy of note is that 92.2% of babies were initiated to breastfeeding and equally received colostrum; meanwhile, 7.8% of babies were not initiated to breastfeeding and as a result did not equally receive colostrum.

**Table 3:** Characteristics of factors that affected the duration of EBF.

Variable	Level	Number	*n* (%) practiced EBF	Statistical significance (*P*)
Employment	Employed (formal/informal)	273	100 (36.6%)	0.425
	Unemployed	230	91 (39.6%)	

Education level	Basic/no formal education	156	74 (47.4%)	0.005
	Tertiary or higher level	347	117 (33.7%)	

Marital status	Married/cohabitating	356	147 (41.3%)	0.003
	Not married	147	44 (29.9%)	

Abbreviations: EBF: exclusive breastfeeding; P: *P* value (statistical significance). Note: This study found that a nursing mother's employment status did not significantly influence EBF duration (*P* value =0.425>5%).

**Table 4:** Final Cox proportional hazard model with factors affecting duration of exclusive breastfeeding in Yaoundé, Cameroon.

Term	Hazard ratio	95% CI	Coefficient (*β*)	S.E	*Z*-statistic	*P*-value
Tertiary/basic education	1.43	1.11-1.84	0.3586	0.1844	2.78	0.005
Married/not married	0.70	0.55-0.89	-0.3525	0.8479	-2.92	0.003
Employed/unemployed	1.12	0.85-1.48	0.1143	0.1607	0.80	0.425

Abbreviations: 95% CI: confidence interval; SE: standard error; P: *P* value (statistical significance). Note: Marital status was negatively correlated to EBF practices (Coef = -0.353) as represented in Table 4.

## Data Availability

The raw dataset used to support this finding are available for this study and can be provided by the corresponding author upon request.
